# Tracing the development of primary students' expressed sustainability conceptions through SDG-based classroom action research

**DOI:** 10.3389/fpsyg.2026.1944639

**Published:** 2026-09-18

**Authors:** Ahmet Akif Erbaş, Tolga Topcubaşı

**Affiliations:** 1 Department of Child Care and Youth Services, Ahmet Erdoğan Vocational School of Health Services, Zonguldak Bülent Ecevit University, Zonguldak, Türkiye; 2 Department of Primary Education, Faculty of Education, İstanbul 29 Mayıs University, İstanbul, Türkiye

**Keywords:** classroom action research, Education for Sustainable Development, primary education, sustainability literacy, Sustainable Development Goals

## Abstract

This qualitative classroom action research study traced the development of primary school students' expressed sustainability conceptions during an 8-week interdisciplinary process based on the Sustainable Development Goals (SDGs). Participants were 31 fourth-grade students (aged 9–10) in one intact classroom of a public primary school in Istanbul, Türkiye. Instruction comprised 24 class periods of 40 min across eight weekly action research cycles; the second author taught all lessons while the first author observed every session. Data comprised pre- and post-instruction conception tasks, learning artifacts, activity-specific assessment forms, audio-recorded and verbatim-transcribed dialogue, observation records, reflective journals, and field notes, analyzed through codebook thematic analysis, criterion-referenced categorization, and a matched participant-level comparison using one common six-domain codebook at both time points. Before instruction, students' responses were dominated by everyday routines, literal notions of continuity, and isolated environmental behaviors; post-instruction responses were broader and more differentiated, the mean number of domains expressed rising from 1.58 to 3.19. Environmental content rose from 10 to 30 of the 31 students and social and ethical content from 4 to 23; resource use and future-oriented outlook, absent from every initial response, appeared in 25 and 14 final responses; and everyday-routine framing, initially expressed by 23 students, appeared in none. Responses also became longer, and expressed domains grew in almost the same proportion, so domain density was essentially unchanged (19.5 vs. 18.5 domains per 100 words): the shift the data support concerns the kind of content expressed, not its density. Relational reasoning—for example, connecting drought with agricultural production, food prices and hunger—was observed in selected dialogue episodes rather than measured across the class, and development was not uniform. Classroom evidence, including misconceptions, prompted five documented instructional adaptations, four of them within lessons. Within this classroom, the post-instruction responses were broader and more multidimensional, understood here as one dimension of emerging sustainability literacy. The findings illustrate how SDG-based, dialogic, and iteratively refined teaching can accompany such broadening. Whether conceptual change of this kind contributes to longer-term nature connectedness or environmental commitment was not examined here and is identified as a direction for subsequent research.

## 1 Introduction

The growing complexity of contemporary environmental, social and economic challenges has made sustainability a defining concern of the 21st century. Climate change, biodiversity loss, resource depletion, unsustainable consumption and social inequality cannot be understood as isolated problems; they require individuals to recognize relationships among ecological systems, social structures, economic decisions and the wellbeing of future generations (Le Blanc, 2015). The United Nations 2030 Agenda introduced the 17 Sustainable Development Goals (SDGs) as a global framework for advancing human wellbeing while protecting the ecological foundations on which societies depend (United Nations, 2015). Education is accordingly expected not only to transmit knowledge about sustainability challenges but to prepare learners to understand complexity, evaluate alternatives and make responsible decisions (UNESCO, 2020; Vladimirova and Le Blanc, 2016).

Education for Sustainable Development (ESD) is the central educational response to these expectations. UNESCO defines it as an approach equipping learners with the knowledge, skills, values and agency needed to act for environmental integrity, economic viability and social justice (UNESCO, 2017, 2020). Rather than treating sustainability as a discrete topic, ESD promotes learning that questions assumptions, explores interdependencies and engages with uncertainty (Boström et al., 2018). Within this perspective the SDGs provide a meaningful pedagogical context, linking environmental issues with poverty, health, equality, consumption, peace and partnership, and enabling students to move beyond narrow environmental interpretations (Vladimirova and Le Blanc, 2016; UNESCO, 2017; O'Flaherty and Liddy, 2018).

Against this background, sustainability literacy has become an important educational outcome (Wang and Guo, 2026). Although conceptualized in different ways, it is generally understood as the capacity to understand sustainability challenges, recognize relationships within socio-ecological systems, evaluate alternatives, consider future consequences and contribute to informed action, integrating knowledge, values, systems thinking, futures thinking, collaboration and action competence (Vesterinen and Ratinen, 2024; Sass et al., 2020; Wiek et al., 2011; Brundiers et al., 2021; Redman and Wiek, 2021). In the present study sustainability literacy is retained as the broad theoretical horizon of this literature, while the empirical construct examined is deliberately narrower: the study investigates students' expressed conceptual understandings of sustainability—what students say and write about the concept—as one dimension of emerging sustainability literacy.

Several components of the broader construct accordingly fall outside what this design can address. Application of sustainability knowledge in authentic out-of-school situations, participation in collective decision-making beyond the classroom, critical action, and sustained collaboration were neither taught as separate objectives nor assessed; where students refer to acting, helping, or conserving, these are expressed intentions recorded in classroom talk and writing. The study therefore speaks to the conceptual dimension of the construct and is silent on its enactment.

This aligns with GreenComp, which organizes sustainability competence into embodying sustainability values, embracing complexity, envisioning sustainable futures and acting for sustainability (Bianchi et al., 2022; Toma et al., 2024). GreenComp is used here not as a standardized assessment framework but as a secondary interpretive lens for examining whether students' evolving conceptions reflect increasingly sophisticated thinking; the analytical focus is on how conceptions develop through authentic classroom experience, not on whether children demonstrate predefined competencies (Vesterinen and Ratinen, 2024).

Primary education is a particularly important context for such development, because children arrive with prior experiences, values and socially constructed understandings that shape how they interpret sustainability-related learning (Andersson and Öhman, 2017). These initial conceptions are often partial, yet they are valuable starting points for conceptual growth (Petrou and Korfiatis, 2022). Walshe (2008) showed that students' understandings frequently reflect incomplete interpretations of the relationships underlying sustainable development, and more recent work indicates that classroom dialogue enables young learners to express, negotiate and reconsider their ideas (Rodnes and Dolonen, 2023; Reffhaug et al., 2024). Prior conceptions are therefore better treated as pedagogical resources than as errors to be corrected (Hedefalk et al., 2014; Erbaş et al., 2026).

Reviews of environmental and sustainability education report positive effects on students' knowledge, attitudes and behaviors (Ardoin et al., 2018; Wetering et al., 2022; Hedefalk et al., 2015). They also identify a limitation: such studies show that interventions can be effective, but say comparatively little about how students' understandings evolve during instruction or how teaching responds to emerging misconceptions. Reviews of competence assessment reach a similar conclusion, no single approach capturing the multidimensional nature of sustainability learning (Redman et al., 2021). Research is therefore needed on the pedagogical processes through which such learning develops, and not only on its outcomes (O'Flaherty and Liddy, 2018).

A persistent difficulty concerns the relationship between predetermined instructional design and students' actual learning needs. Teachers may begin with clearly specified goals, yet students' responses reveal misunderstandings, alternative interpretations and unanticipated connections (Öhman and Öhman, 2013; Petrou and Korfiatis, 2022). A fixed design records these as outcomes without allowing them to influence subsequent instruction (Hedefalk et al., 2015). Classroom action research, by contrast, provides a framework in which teaching is continuously informed by classroom evidence through cycles of planning, action, observation, reflection and revision (Reffhaug et al., 2024), a logic particularly compatible with a field in which complexity, dialogue and critical reflection are themselves dimensions of learning (Haggström, 2022).

Three related gaps remain (O'Flaherty and Liddy, 2018; Vesterinen and Ratinen, 2024). Much of the evidence concerns outcomes such as knowledge, attitudes or predefined competencies rather than how children's initial conceptions are reorganized (Redman et al., 2021; Bascope et al., 2019). Studies frequently report pre- and post-intervention differences while offering less detail about the pedagogical decisions taken between those two points (Sinakou et al., 2019). And classroom-based action research documenting the relationship between iterative instructional refinement and change in students' expressed conceptions remains limited (Haggström, 2022).

Against this background, the present study employs classroom action research to trace the development of primary school students' expressed sustainability conceptions during an 8-week interdisciplinary SDG-based instructional process. The conceptual logic of the study is deliberately simple (Figure 1): Education for Sustainable Development provides the overarching educational orientation; the SDGs function as interdisciplinary pedagogical contexts rather than as the learning outcome itself; classroom action research constitutes the iterative methodology through which instruction is refined; GreenComp serves as a secondary interpretive lens; and students' expressed sustainability conceptions constitute the direct empirical outcome. Because lifelong nature connectedness, environmental commitment, and other long-term psychological outcomes were not measured, the study makes no empirical claims about them; its educational-psychological contribution is grounded instead in classroom-bounded processes supported by the data—elicitation of prior conceptions, conceptual differentiation, guided dialogue, relational reasoning, and reflective instructional adaptation—which may provide a foundation upon which such longer-term outcomes can later be built (Chawla, 2020; Wells and Lekies, 2006).

**Figure 1:**
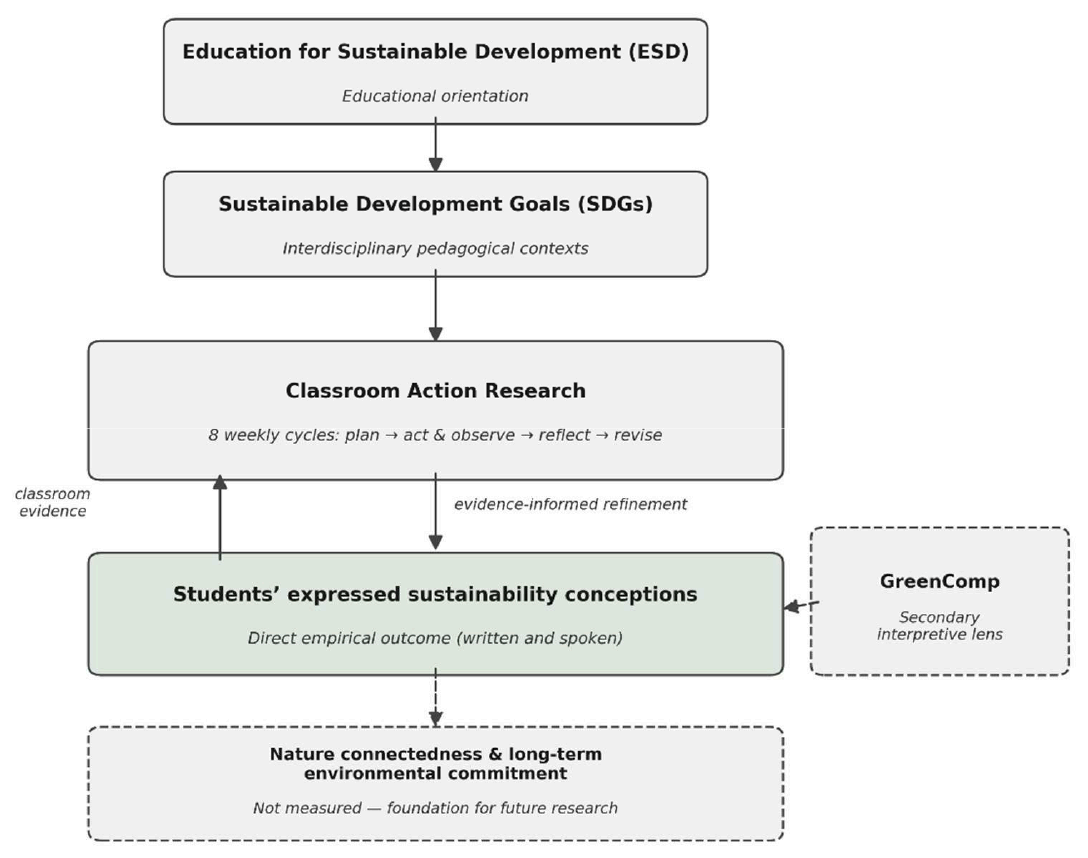
Conceptual logic of the study. Long-term nature connectedness and environmental commitment are shown as an unmeasured horizon for future research.

Accordingly, the primary purpose of this study was to trace how primary school students' expressed sustainability conceptions changed during an interdisciplinary SDG-based instructional process, and its secondary, methodological purpose was to document how that process was iteratively designed, evaluated, and refined in response to classroom evidence. The study's primary contribution is therefore an account of conceptual development in students' own written and spoken expressions; the documentation of instructional refinement is reported as a secondary contribution that helps to contextualize, but does not explain, that development. Specifically, the study addressed the following research questions:

RQ1. How did primary school students conceptualize sustainability before participating in the SDG-based instructional process?

RQ2. How was the SDG-based instructional process iteratively refined in response to classroom observations, student responses, and researcher reflections throughout the action research process?

RQ3. How did students' expressed sustainability conceptions change over the course of the eight-week instructional process, at both the group level and the matched participant level?

RQ4. What evidence from students' learning artifacts, classroom dialogue, observations, reflective journals, and field notes illustrates these conceptual changes and contextualizes them?

The study contributes to the literature in three ways. It shifts attention from sustainability awareness as an isolated outcome to the development of students' expressed conceptions, examined at group and matched participant levels; it treats the SDGs as a pedagogical framework for interdisciplinary learning rather than as instructional content; and it documents the iterative pedagogical decisions through which instruction was refined, together with the classroom evidence on which each rested.

## 2 Method

### 2.1 Research design

This study employed a classroom-based action research design to investigate how an interdisciplinary instructional process based on the Sustainable Development Goals (SDGs) was accompanied by changes in primary school students' expressed sustainability conceptions. The design followed the cyclical model of planning, action, observation, reflection, and revision described by Kemmis et al. (2014). The study is described as researcher-led classroom action research and not as critical participatory action research: the research team designed and taught the sequence, the class's regular teacher did not take part in the cycles, and the students were participants and not co-inquirers. The spiral is therefore adopted here as a procedural model for evidence-informed instructional revision, not as a claim to the participatory commitments that define the critical tradition. Each instructional week constituted one action research cycle, so that eight successive cycles were completed. For every cycle, the instructional intention, the classroom evidence collected, the researchers' reflective interpretation, the resulting pedagogical decision, the actual modification implemented, and the subsequent evidence were documented in a structured cycle log (Table 1). Rather than treating instruction as a fixed intervention, the study conceptualized teaching as an adaptive process that evolved through these iterative cycles.

**Table 1:** Documentation of the eight weekly action research cycles.

Week/ cycle	Instructional intention	Classroom evidence	Reflective interpretation	Pedagogical decision and actual modification	Subsequent evidence
1	Introduce poverty and hunger through role allocation, station work, and empathy-based storytelling	Students explained hunger as a simple lack of food; several spontaneously described family crop failures caused by drought (transcript)	Everyday experiences could anchor relational reasoning about the causes of hunger	Immediate adaptation: extended the discussion with guided questions linking drought, irrigation, production, prices, and access to food	Students articulated multi-step links among climate, agriculture, prices, and hunger; station worksheets framed poverty as deprivation of basic rights
2	Explore ecosystems, climate action, and life below water and on land through map-based group work and role-based empathy tasks	In written role-taking tasks students framed pollution as a direct threat to the vital functions of living beings (e.g., impaired breathing, habitat loss, infection from discarded batteries)	Students already reasoned about ecological harm in causal and ethical terms; the affective framing was productive rather than in need of correction	No substantive modification required; the planned sequence proceeded, and the affective framing was deliberately retained and built upon in later weeks	Artifacts showing causal chains (waste → reduced oxygen → death) and expressions of moral indignation toward human-caused pollution
3	Water filtration experiment on clean water, health, and the nature of science	Of the 27 students present, 13 framed water safety solely in terms of removing visible impurities and 9 proposed no treatment at all; only 5 referred to a biological step (pre-activity forms)	A shared misconception equating visual clarity with potability	Immediate adaptation: explicit contrast between visible cleanliness and biological safety, with discussion of microorganisms and boiling; prospective adaptation: revised post-activity prompt targeting the function of filter materials	Dialogue distinguishing microbes from visible dirt, and responses to the new post-activity prompt on filter-material function (14 qualified, 8 intermediate, 5 limited against that prompt's own criteria, which are not those applied before the activity)
4	Fictional-country scenario on international cooperation and partnerships	Several students proposed that a single militarily powerful country should solve the crisis alone (transcript)	Single-actor, single-solution reasoning about a multidimensional crisis	Immediate adaptation: a sequence of counter-questions addressing hunger, unemployment, and infrastructure separately	Students proposed complementary multi-actor contributions (“one with soldiers, one with money, one with technology”)
5	Narrative-based activity on educational access, gender equality, and reduced inequalities	Initial contributions consisted largely of simple assent to equality statements (transcript)	Support for equality expressed without justification, with a risk of socially desirable responding	Immediate adaptation: prompts requiring justification (“Should people's abilities be judged according to whether they are girls or boys?”) and a follow-up question inviting students to imagine being in the position of an unsupported peer, which elicited concrete proposals	Students produced fairness-, opportunity-, and potential-based justifications and concrete support plans
6	Justice-related scenarios, institutional analysis, and classroom discussion	Students discussed institutional responsibility and fairness in the planned scenarios; the cycle log records no misconception or unexpected response calling for a change of plan	The planned scenarios were functioning as intended	No substantive modification; the planned activities proceeded as designed	Discussion proceeded as planned; no cycle-specific outcome separable from the overall sequence was recorded
7	Energy-source analysis and responsible consumption tasks	Students analyzed energy sources and consumption habits in group tasks; the cycle log records no misconception or unexpected response calling for a change of plan	The planned tasks were functioning as intended	No substantive modification; the planned activities proceeded as designed	Group tasks completed as planned; no cycle-specific outcome separable from the overall sequence was recorded
8	Analysis and redesign of an unsustainable city	Group designs drew on renewable energy, recycling, biodiversity, and water management; reflective journal recorded deliberate rather than random design choices	An opportunity for cumulative integration of concepts introduced in earlier weeks	Preplanned cumulative task (see Table 2): groups were required to connect concepts from earlier weeks and justify each design choice. Not counted as an evidence-driven adaptation	Designs and explanations linking industrial activity, air quality, environmental quality, and human wellbeing

The eight implementation cycles were preceded by a structured preparatory phase: a needs analysis; a review of the relevant literature and of the fourth-grade curriculum; alignment of selected SDGs with specific curriculum outcomes; design of the instructional activities, scenarios, and materials; external expert review of the plan and materials, followed by revision in line with the feedback received; and administration of the initial sustainability conception task. The SDG-curriculum alignment drew on fourth-grade outcomes in social studies, science, and human rights and civics education (e.g., IHVD.4.1.3 on interpreting fundamental human rights; SB.4.1.3 on generating ideas for sustaining social cohesion; FB.4.1.1 on reflecting on the nature of science; FB.4.6.2 on critical thinking about renewable and non-renewable energy; SSB.4.5.3 on analyzing production, distribution, and consumption processes; and FB.4.8.1 on scientific inquiry into building a sustainable living environment).

### 2.2 Research context and participants

The study was conducted during the 2024–2025 academic year in a public primary school in Istanbul, Türkiye. The school and the class were selected through convenience sampling, on the basis of accessibility and the willingness of the school administration and the classroom teacher to host the 8-week process. The school is located in the Ataşehir district of Istanbul; its socioeconomic profile was established by examining the parental income and occupation information held in the school's own enrolment records, on the basis of which the student population was characterized as predominantly middle socioeconomic status. Participants were 31 fourth-grade students (17 girls, 14 boys) from one intact classroom, aged 9–10 years. The language of instruction and of all data collection was Turkish. Students had received no prior formal instruction specifically addressing the SDGs, although environment-related topics form part of the standard fourth-grade curriculum. All 31 eligible students in the class were invited, parental consent was obtained for all of them, and no student or dataset was excluded from the analysis.

The intervention comprised three class periods per week over eight consecutive weeks, giving 24 class periods in total. Each period lasted 40 min, so the process amounted to 16 clock hours of instruction; “session” refers throughout to one such class period. All 31 students took part in the instructional process as a whole, although short-term absences occurred in individual sessions. In particular, four students were absent during the third-week filtration activity, so the valid sample for that activity-specific assessment was 27. A control group was not included because the purpose of the study was not to determine causal effectiveness but to examine students' expressed sustainability conceptions and the iterative refinement of instruction within a classroom action research framework.

To protect confidentiality, the school's name and all identifying information were removed. Student responses and quotations were anonymized using participant codes.

### 2.3 Research team and researcher role

The research team comprised two researchers who collaboratively designed, evaluated, and refined the instructional process. Responsibilities during implementation were divided explicitly: the second author (hereafter the teacher-researcher) implemented all 24 instructional sessions, while the first author (hereafter the observing researcher) was present in every lesson as a non-participant observer responsible for systematic observation and record keeping. The class's regular teacher was not present during the implementation sessions and did not deliver any part of the instruction. The teacher-researcher is an assistant professor of primary education with 12 years of experience as a primary school teacher; the observing researcher is an associate professor with 15 years of professional experience in the field of child development and education; and the team's prior relationship with the school was limited to obtaining permission for this study, with no prior institutional or professional connection. In the dialogue excerpts reported in the Results, the speaker labeled “Teacher-researcher” is the second author.

This division of roles addressed a key methodological concern of practitioner research: because the teacher-researcher could not simultaneously teach and observe systematically, structured observation was carried out in every session by the observing researcher, using an observation protocol focused on students' verbal responses, misconceptions, emerging conceptual connections, participation, questions, and peer interaction. In addition, all lessons were audio-recorded, so that classroom dialogue could be transcribed verbatim and observational records could be checked against the recordings after each session. The teacher-researcher added complementary reflective notes immediately after each lesson. At the end of each weekly cycle, the two researchers jointly reviewed the accumulated evidence—student responses, transcripts, observation records, reflective journals, field notes, and learning artifacts—to examine students' emerging understandings, identify conceptual challenges, and determine whether subsequent instruction required modification. These reflective discussions, and the decisions arising from them, were recorded in the cycle log (Table 1).

Because the researchers designed the intervention, implemented the teaching, and conducted the analysis, reflexivity was treated as a central methodological concern rather than a formality. Both researchers entered the study expecting the instructional process to broaden students' conceptions, and this expectation itself was documented and interrogated. Reflective journals were used not only to record student learning but also to make the researchers' pedagogical decisions, theoretical assumptions, and interpretive hunches explicit; weekly debriefings deliberately sought disconfirming evidence. In practice this yielded two kinds of finding, both reported in the Results: students whose responses changed only narrowly, and written responses that continued to reproduce the reasoning a lesson had been designed to unsettle. The record contains no documented instance of an activity abandoned as unworkable, and no disagreement between the two researchers that survived to the point of decision. The absence of recorded disagreement between two closely collaborating researchers is treated here as a limitation of the procedure, not as evidence of analytic convergence.

### 2.4 Action research process and cycles

The sequence was designed to progress from relatively concrete and experience-based sustainability issues toward more complex, interconnected, and future-oriented challenges. Early weeks focused on poverty, hunger, health, clean water, environmental protection, and basic human needs. Later activities addressed cooperation, equality, justice, energy, responsible consumption, production systems, innovation, and sustainable cities.

While Table 2 presents the preplanned instructional sequence, Table 1 documents the action research cycles as they actually unfolded: for each weekly cycle, the initial instructional intention, the classroom evidence that emerged, the researchers' reflective interpretation, the pedagogical decision taken, the actual modification implemented, and the subsequent evidence observed. It shows which changes were introduced in response to classroom evidence and which belonged to the planned movement from concrete to complex topics, and it records the cycles in which the evidence called for no modification.

**Table 2:** Overview of the 8-week SDG-based interdisciplinary instructional process.

Week	SDGs addressed	Main learning activities	Pedagogical focus
1	SDG 1: No poverty; SDG 2: Zero hunger	Role allocation, station activities, empathy-based storytelling, collaborative discussion	Basic needs, poverty, human rights, empathy, and social responsibility
2	SDG 13: Climate action; SDG 14: Life below water; SDG 15: Life on land	Map-based group work, environmental problem solving, solution matching	Relationships among ecosystems, living organisms, environmental problems, and human actions
3	SDG 3: Good health and wellbeing; SDG 6: Clean water and sanitation	Water filtration experiment, written tasks, observation, classroom discussion	Physical cleanliness, biological safety, and scientific reasoning
4	SDG 17: Partnerships for the goals	Fictional country scenario, role representation, collaborative decision-making	Interdependence, cooperation, and shared responsibility
5	SDG 4: Quality education; SDG 5: Gender equality; SDG 10: Reduced inequalities	Narrative-based activity, discussion of access and inequality	Equality of opportunity, gender equality, social justice, and inclusion
6	SDG 16: Peace, justice and strong institutions	Justice-related scenarios, institutional analysis, classroom discussion	Justice, peace, social trust, civic awareness, and institutional responsibility
7	SDG 7: Affordable and clean energy; SDG 12: Responsible consumption and production	Energy-source analysis, discussion of consumption, collaborative tasks	Energy literacy, responsible consumption, resource use, and citizenship
8	SDG 8: Decent work and economic growth; SDG 9: Industry, innovation and infrastructure; SDG 11: Sustainable cities and communities	Analysis and redesign of an unsustainable city	Systems-oriented reasoning, future thinking, urban sustainability, and integration of prior learning

The weekly activities were implemented through iterative action research cycles in which classroom evidence informed subsequent instructional decisions. At the end of each weekly cycle, the researchers reviewed student responses, classroom dialogue, learning artifacts, observation records, and reflective notes to identify emerging understandings, misconceptions, and learning needs. The decisions taken, including the cycles in which the evidence called for no change, were recorded in the cycle log.

Instructional adaptations were classified using explicit criteria. Immediate adaptations were changes enacted during an ongoing lesson in direct response to student contributions; prospective adaptations were changes made to a later task or lesson on the basis of evidence reviewed within the weekly cycle; and cumulative refinements were deliberate decisions to revisit and integrate previously introduced concepts in later, more complex tasks. Across the eight cycles, five substantive adaptations were documented: four immediate and one prospective, all involving changes to lesson content or to the questions asked. The eighth-week task appears in Table 1 as a cumulative refinement but is not counted among them, since Table 2 already lists integration of prior learning as its preplanned focus. Only one adaptation, the revised third-week prompt, altered a later task rather than the lesson in progress; none carried across weekly cycles, and the remaining four were within-lesson responses, which is responsive teaching and not by itself evidence of a developmental spiral.

### 2.5 Data collection sources

Data were collected from initial and final sustainability conception tasks, student learning artifacts, activity-specific assessment forms, structured classroom observations, audio-recorded and transcribed dialogue, reflective journals and field notes, enabling comparison across students' written, verbal, observational and reflective evidence. Table 3 inventories the corpus, reporting for each source its timing, unit, number of items, participating students, language, recording format and missing data; it is reproduced as Supplementary Table S4. A session-by-session inventory, together with an explicit statement of which counts were and were not kept as separate registers during fieldwork, is provided as Supplementary Tables S5, S6.

**Table 3:** Inventory of the qualitative data corpus.

Data source	Timing	Individual/group	Items or episodes	*n*	Language	Recording format	Missing data
Initial conception task	Before instruction	Individual, written	31 responses	31	Turkish	Handwritten forms	No records lost
Final conception task	After instruction	Individual, written	31 responses	31	Turkish	Handwritten forms	No records lost
Water activity pre-assessment	Week 3	Individual, written	27 responses	27	Turkish	Written forms	4 students absent from the session
Water activity post-assessment	Week 3	Individual, written	27 responses	27	Turkish	Written forms	4 students absent from the session
Student learning artifacts	Weeks 1–8	Individual and group	8 activity sets, one per week	31	Turkish	Worksheets, role-taking tasks, scenario responses, design products	Attendance varied by session
Classroom dialogue	Weeks 1–8	Whole class/group	24 recorded lessons; 4 episodes meeting the selection criteria	31	Turkish	Audio recording; verbatim transcription	No recording loss; attendance varied by session
Structured observation records	Weeks 1–8	Whole class	24 session records	31	Turkish	Structured protocol	No records lost; attendance varied by session
Reflective journals	Weeks 1–8	Researchers (2)	16 weekly entries	–	Turkish	Written journals	No records lost
Field notes	Weeks 1–8	Researchers (2)	24 sets of notes (one per session)	–	Turkish	Written notes	No records lost

The sources contributed in different ways, and not all are equally open to inspection. The conception tasks, the activity-specific forms, the transcripts, the student artifacts and one reflective-journal entry are quoted in the Results, so their evidential basis is open to scrutiny. The structured observation records and the field notes are not quoted: they functioned as working documents that fed the weekly cycle meetings, informed the classroom-evidence column of Table 1, and guided the identification of candidate episodes for transcription. They are represented here by their analytic product and not by quoted material, so claims resting on them—principally those concerning instructional refinement—are less open to independent appraisal than claims resting on the quoted sources.

#### 2.5.1 Initial and final sustainability conception tasks

The same open-ended conception task was administered before and after the 8-week process, designed to identify the meanings students themselves attributed to sustainability rather than to test reproduction of a definition. The initial responses provided a baseline; the final responses allowed examination of whether conceptions had broadened toward resource conservation, responsible consumption, protection of living organisms, social responsibility, equality and future generations.

#### 2.5.2 Student learning artifacts and activity-specific assessments

Selected activities also included activity-specific assessment forms. The third-week water-filtration activity was selected as the principal assessment example on documentary grounds: of the 8 weeks, only that activity generated structured written responses from every student present both before and after the task, so it is the only activity for which criterion-referenced categorization could be applied to two separate written datasets. All student learning artifacts produced during the 8 weeks were reviewed and coded, and individual and group products were distinguished during analysis. The artifacts were archived by weekly activity set rather than itemized in a running register, so the corpus is reported here as eight activity sets (Table 3) and not as a total count of individual and group products. Students used a commercially available multi-stage water-filtration kit and the sequence was taught through a demonstration-and-practice technique: the teacher-researcher modeled each step while the students assembled and operated their own filters simultaneously, so that every student present completed the procedure through direct experience. Before the filtration activity, students were asked:

“If you had to drink water from a puddle, how would you clean it?”

Responses were examined in relation to physical treatment awareness and biological safety awareness. After the activity, students responded to a prompt revised in the light of their pre-activity answers (Table 2):

“Would the result be the same if only one material had been used in the filter? Why?”

#### 2.5.3 Classroom observations and dialogue

Observation followed the protocol described above and was carried out in every session by the observing researcher. The records served both to identify evidence of learning and to inform instructional refinement, and were cross-checked against the audio recordings after each lesson.

All lessons were audio-recorded under the written parental consent obtained for the study, which explicitly covered recording, and classroom dialogue was transcribed verbatim in Turkish. The dialogue excerpts presented in this article were drawn from these transcripts, anonymized using participant codes, translated into English independently by the two researchers, and reconciled by consensus to check semantic equivalence. Episodes were treated as candidate critical learning episodes when they met three criteria: (a) a student idea or misconception became explicitly visible; (b) the idea was challenged, questioned, or extended in interaction; and (c) subsequent talk or written work indicated reconsideration or elaboration. The full set of transcripts from all 24 lessons was screened against these criteria, and four episodes—from weeks 1, 3, 4, and 5—met all three; these four are the episodes reported in the Results, and no qualifying episode that contradicted the reported patterns was excluded. Screening proceeded transcript by transcript rather than through a separate log of provisionally marked exchanges, so the number of exchanges considered before the criteria were applied cannot be reported retrospectively. This assurance is weaker than it appears. Because criterion (c) refers to an outcome, an exchange in which an idea was raised and challenged but not subsequently reconsidered could not satisfy the criteria and would not enter the qualifying set. The selection procedure is therefore biased toward episodes in which something changed, and the four reported episodes should be read as illustrations of productive interaction rather than as a representative sample of classroom talk. The dialogue excerpts are presented in condensed form: turns that did not bear on the conceptual issue under discussion have been omitted for length, and every such omission is marked in the excerpt with a bracketed ellipsis. These dialogues were analyzed as evidence of conceptual expression and negotiation rather than used merely as illustrative quotations.

#### 2.5.4 Alignment between research questions and data sources

Table 4 sets out which sources served as primary and supporting evidence for each research question. Taken together, these data sources enabled the study to examine students' expressed sustainability conceptions as a developing and multidimensional outcome. The initial and final conception tasks documented differences in students' expressed conceptions; classroom dialogue, artifacts, observations, activity-specific assessments, journals, and field notes contextualized those differences and recorded how instruction was revised in response to classroom evidence.

**Table 4:** Alignment between research questions and data sources.

Research question	Primary data sources	Supporting data sources
RQ1. How did students conceptualize sustainability before the SDG-based instructional process?	Initial sustainability conception task	Initial artifacts and field notes
RQ2. How was instruction iteratively refined in response to classroom evidence?	Reflective journals and observation records	Student responses, dialogue, artifacts, and field notes
RQ3. How did students' expressed sustainability conceptions change over the 8-week process?	Initial and final conception tasks and learning artifacts	Activity-specific assessments, dialogue, and observations
RQ4. What evidence illustrates these conceptual changes?	Artifacts, dialogue, observations, and researcher journals	Activity-specific assessments and field notes

### 2.6 Data analysis

Data were analyzed through a multi-layered qualitative approach designed to examine both changes in students' expressed sustainability conceptions and the iterative refinement of instruction. Three analytical approaches were deliberately used for different datasets. First, the conception tasks, the dialogue transcripts and the student learning artifacts were analyzed through codebook thematic analysis: coding followed the phases described by Braun and Clarke (2006), but a shared codebook and consensus procedures were adopted rather than a purely reflexive orientation (Braun and Clarke, 2019). Second, the activity-specific assessments were classified through criterion-referenced qualitative categorization using predefined checklists. Third, the action research process was examined through documentary analysis of the cycle log, observation records, and reflective journals. In addition, a matched participant-level cross-time analysis linked each student's initial and final conception-task responses, as described below.

#### 2.6.1 Analysis of initial and final sustainability conceptions

Students' responses to the open-ended sustainability conception task, administered before and after the intervention, were analyzed using codebook thematic analysis. The analysis proceeded in six steps: familiarization with the full set of responses; independent initial coding of the 31 initial and 31 final responses by both researchers; consolidation of initial codes into a shared codebook with definitions and example excerpts; independent application of the codebook to the full datasets; comparison of codings and resolution of disagreements by returning to the original Turkish responses; and organization of consolidated codes into themes. Initial and final datasets were first coded separately to avoid imposing categories emerging from the post-instruction responses onto students' initial conceptions. Following these separate analyses, the datasets were compared to identify patterns of continuity, expansion, disappearance, and emergence. Because the two codebooks were not symmetrical, this stage supported description of each time point but not direct cross-time comparison; the recoding described below was carried out for that purpose.

Code frequencies were reported descriptively to indicate the prominence of emerging themes. For each frequency reported, the denominator is the number of students who completed the corresponding task (*n* = 31). A coded idea was defined as one codebook category applied to one response: a category was counted once per response however often the underlying idea recurred in it, so that a student's idea count equals the number of distinct categories present in that student's answer. To illustrate the segmentation, the initial response “Getting ready in the morning and going to school, eating, and doing homework” was assigned two categories—school and study routines, and basic needs and self-care—and therefore counted as two coded ideas, whereas the same student's final response was assigned five. On this basis the initial dataset yielded 61 coded ideas and the final dataset 121, corresponding to means of 1.97 and 3.90 per student. Frequencies were interpreted descriptively rather than as evidence of causal effectiveness.

#### 2.6.2 Matched participant-level cross-time analysis

The inductive analyses reported above produced two separate codebooks, one for each time point, and these are not symmetrical: no everyday-routine code was generated for the final dataset, and no resource-use or future-oriented code for the initial dataset. A comparison built on them would therefore confound genuine change with the structure of the coding frames. To avoid this, both sets of responses were recoded from the original scripts using a single common codebook of six domains, applied identically at the two time points: everyday routines and personal wellbeing; continuity of life or practice; environmental care; social and ethical responsibility; resource use and consumption; and future-oriented and global outlook (Supplementary Table S2 gives the definitions and anchor examples). Action-related content was not made a separate domain, since in these responses it was expressed through the environmental-care and resource-use categories rather than independently of them; it is reported at group level only.

Continuity was retained as a domain in its own right rather than folded into the everyday category, because the Turkish term for sustainability is morphologically derived from the verb “to continue” and the question of whether students abandon or reinterpret that sense is substantive rather than incidental. A response was assigned to the continuity domain when it framed sustainability as something being kept going, irrespective of what was to be kept going; this makes it possible to distinguish an empty literal use from one filled with sustainability content.

Each student's initial and final responses were then represented as the set of domains expressed, and for every student it was recorded which domains were added, retained, or no longer expressed. Trajectories were classified as expansion (domains added, none lost), replacement (domains both added and lost), contraction (domains lost, none added), or stability. Both researchers coded independently and resolved differences by returning to the original Turkish scripts. To guard against interpreting longer answers as richer thinking, the number of words per response and the number of domains expressed were also counted, and domain density was compared across time. Results are reported in the Results (Matched Participant-Level Trajectories), in Table 5, and at participant level in Supplementary Table S3.

**Table 5:** Matched cross-time analysis using a common six-domain codebook (*N* = 31).

Domain	Expressed initially (*n*)	Expressed finally (*n*)	Added (*n*)	Retained (*n*)	No longer expressed (*n*)
D1 Everyday routines and personal wellbeing	23	0	0	0	23
D2 Continuity of life or practice	12	7	4	3	9
D3 Environmental care	10	30	21	9	1
D4 Social and ethical responsibility	4	23	19	4	0
D5 Resource use and consumption	0	25	25	0	0
D6 Future-oriented and global outlook	0	14	14	0	0

Both time points were recoded from the original scripts with the same codebook, so all six rows are directly comparable. “Retained” counts students expressing the domain at both time points. The initial continuity count differs from the inductive frequency in Table 6 because the common codebook defines this domain more broadly. Because that codebook was constructed after the inductive coding, the two domains absent from every initial response may partly reflect categories generated from the final data. Definitions and anchor examples are given in Supplementary Table S2; participant-level trajectories in Supplementary Table S3.

Classroom dialogue was examined to identify critical learning episodes in which students' ideas became visible, were challenged, reconsidered or expanded through interaction; each was analyzed with respect to the initial understanding, the exchange that followed, and the evidence of broadening it contained.

**Table 6:** Students' initial conceptions of sustainability.

Theme	Codes	*f*
Everyday life and educational routines	Going to school, studying, and doing homework	14
	Basic needs and self-care	9
	Social activities and play	7
Environmental awareness	Waste management and recycling	4
	Keeping the environment clean	3
	Protecting nature and living organisms	3
Conceptual meanings and life values	Continuity of life and ongoing processes	11
	Psychological wellbeing and a good life	6
	Helping others and maintaining positive social relations	4

#### 2.6.3 Analysis of activity-specific assessments

Pre-activity responses were examined against a two-item checklist: (a) awareness of physical treatment (filtering, settling, or removal of visible impurities) and (b) awareness of biological safety (boiling or otherwise eliminating microorganisms). Responses meeting both criteria were categorized as qualified, those meeting only the physical criterion as intermediate, and those meeting neither as limited.

Post-activity responses were examined against a different two-item checklist: (a) correctly predicting that the outcome would not be the same, and (b) justifying this prediction by referring to the distinct function of individual filter materials. Responses meeting both criteria were categorized as qualified, those meeting only the prediction criterion as intermediate, and those meeting neither as limited. Because the pre- and post-activity prompts addressed different constructs, they were analyzed separately rather than as an equivalent pre/post comparison. Three constructs are distinguished in the reporting. The first, recognition that water may carry contamination that cannot be seen, is indexed by the biological-safety criterion of the pre-activity checklist. The second, the inference that visual clarity does not by itself imply potability, is not equivalent to the first, since a student may name boiling as a procedure without abandoning the assumption that clear water is safe; it is bounded, but not measured, by the intermediate category, since an intermediate response is by definition one that treats the removal of visible material as sufficient. The third, reasoning about the differentiated function of filter materials, is indexed by the post-activity checklist. No instrument targeted the second construct directly, and the evidence bearing on it is accordingly weaker than for the other two. Resulting frequencies were used descriptively as qualitative evidence of changes in students' reasoning rather than as standardized achievement measures.

#### 2.6.4 Analysis of the action research process and instructional refinement

The action research process was analyzed documentarily: the cycle log, observation records and reflective journals were read cycle by cycle to establish what evidence was recorded, what interpretation the researchers placed on it, and whether the plan was changed; the resulting reconstruction is reported in Table 1. Finally, interpretations were compared across the qualitative data sources. Conclusions were developed from converging evidence rather than from isolated quotations, subject to the qualification noted above concerning the observation records and field notes. Because the identifiers used for conception responses, dialogue turns and artifacts were not linked to one another, convergence is demonstrated at the level of themes rather than of individual students.

### 2.7 Trustworthiness and research quality

Trustworthiness was addressed through prolonged engagement across 8 weeks, comparison across the sources inventoried in Table 3, independent coding by both researchers with disagreements resolved against the original scripts, and systematic documentation of every stage from curriculum-SDG alignment to analysis, recorded in the cycle log. No numerical intercoder reliability coefficient was calculated; the procedure is described under Data Analysis. Given the researchers' direct involvement in the instructional process, particular attention was paid to grounding interpretations in documented classroom evidence and to reporting evidence that did not fit the emerging account, and the limits of these safeguards are set out in the Limitations subsection of the Discussion.

#### 2.7.1 Transferability

The findings are context-specific rather than statistically generalizable; the article describes the context, participants, instructional process, researcher roles, data sources and analytical procedures in detail so that applicability elsewhere can be judged.

### 2.8 Ethical considerations

The study followed established ethical principles for research involving children. Ethical approval was obtained from the institutional ethics committee before data collection and written parental consent was secured for all participants.

Students and their parents were informed about the purpose of the study, research procedures, confidentiality, voluntary participation, and the right to withdraw at any stage without adverse consequences. In addition to written parental consent, age-appropriate verbal assent was obtained from the students themselves. The written parental consent form explicitly covered audio recording of the lessons; the recordings were used only to produce the transcripts, were not shared outside the research team, and were stored securely. Because the activities were embedded in regular classroom instruction, taking part in the lessons was part of ordinary schooling; voluntariness applied to the use of students' data, and students could ask for their materials to be excluded at any time without any consequence. The researchers' dual role as instructors and investigators was explained to parents and, in age-appropriate terms, to the students themselves. During the water-filtration activity, students did not drink the filtered water at any point, and the lesson explicitly emphasized that classroom filtration does not produce potable water. All identifying information was removed, participant codes were used when reporting quotations and learning artifacts, and research data were stored securely and used solely for scientific purposes.

## 3 Results

### 3.1 Students' initial expressed conceptions of sustainability

Analysis of the pre-instruction sustainability conception task revealed that students' initial understandings of sustainability were largely grounded in everyday experiences, literal interpretations of continuity, and isolated environmental or social behaviors. The responses were organized into three overarching themes: everyday life and educational routines, environmental awareness, and conceptual meanings and life values. All 31 students completed the initial task; the frequencies in Table 6 are counts of responses containing each code (denominator = 31), and because individual responses could contain more than one meaningful idea, multiple codes could be assigned to a single response. The initial dataset yielded 61 coded ideas in total (*M* = 1.97 per student).

The most frequent codes concerned the continuation of familiar routines: going to school, studying and completing homework (*f* = 14), the continuity of life itself (*f* = 11) and basic needs such as eating and self-care (*f* = 9). Environmental interpretations were present but limited, with waste management and recycling (*f* = 4), keeping the environment clean (*f* = 3) and protecting nature (*f* = 3). A third group of responses was value-oriented, associating sustainability with wellbeing, freedom, helping others and living harmoniously. Taken together, the initial responses were concrete, present-oriented and experience-based; sustainability had not yet become an interconnected concept encompassing resource use, social justice or future generations.

A representative initial response in the everyday-routines theme read: “Sustainability is continuing to go to school, studying, and eating and continuing our life” (S01). For the environmental theme: “Keeping what has been used out of the bin by recycling it, so that it continues” (S21). For the social and ethical strand, the only initial code of its kind: “Helping people and not getting on badly with anyone” (S29). The corresponding final responses of the same three students were, respectively: “Not using water and electricity in vain, not throwing leftover food away, separating the rubbish, and leaving a good place for the children of the future without polluting the environment”; “Reducing food waste is necessary, because there are children going hungry in the world. Everyone should have equal rights, and countries should work so that these problems are fewer in the future”; and “We should protect trees, animals and the places where they live, and help those in difficulty. In this way a better world is left for the future.”

A further consideration concerns language. The Turkish term for sustainability, *sürdürülebilirlik*, is morphologically derived from the verb *sürdürmek* (“to continue, to maintain”). Some literal, continuity-centered initial answers may therefore partly reflect the everyday meaning of the word itself rather than a purely conceptual limitation; this possibility is examined in the Discussion.

### 3.2 Iterative instructional refinement across the action research process

Analysis of the 8-week implementation showed that instruction was refined in response to students' emerging ideas, misconceptions, classroom dialogue, and learning artifacts, and these refinements were documented cycle by cycle in the structured log summarized in Table 1. Across the eight cycles, five substantive adaptations were recorded—four immediate and one prospective—all involving changes to lesson content or to the questions asked. Only one of the five, the revised third-week post-activity prompt, altered a later task rather than the lesson in progress; the other four were within-lesson responses. The eighth-week cumulative task is reported in Table 1 but excluded from this count, since Table 2 shows it was preplanned as an integrative task. At the same time, part of the observed progression—most notably the movement from concrete to more complex topics—followed the preplanned sequence presented in Table 2 and should not be attributed to responsive adaptation.

A representative example occurred during the third-week water activity, where students initially equated clean water with the removal of visible impurities. Classroom evidence prompted researchers to expand the lesson to address invisible microorganisms and biological safety. One student stated:

“First, I would remove the sand or leaves from the water, clean it, and drink it.”

Beyond the water activity, documented adaptations from other weeks included the first-week immediate extension of the hunger discussion toward drought, irrigation, agricultural production, and food prices after students spontaneously introduced family farming experiences; the fourth-week counter-questioning sequence introduced when several students proposed single-actor solutions to a multidimensional crisis; and the fifth-week shift from eliciting agreement with equality statements to requiring justifications, prompted by observing that initial contributions consisted largely of socially desirable assent. The eighth-week sustainable-city task, cumulative though its design was, belonged to the original plan and is not among them. By the final week, students integrated these ideas within sustainable-city designs incorporating renewable energy, recycling, biodiversity conservation, water management, and green infrastructure.

The designs record what was integrated. Working from an image of an unsustainable city, groups identified burning trees, dying animals, polluted seas and factory smoke as the features to be changed, and their redesigned cities incorporated solar and wind installations, cycle paths, recycling points reachable from every part of the city, tree planting and dedicated spaces for animals, together with explicit rules such as a hunting ban. These are group products, reported as evidence of what students selected and justified in a design task rather than as individual conceptions.

The reflective journals provide converging evidence about this final task. One entry recorded:

“Particularly during the sustainable-city design phase, rather than making random choices, the children turned the knowledge they had acquired during the process into deliberate decisions and reflected these in their designs… That topics covered in earlier weeks were carried into the task without being forgotten supports the integrative quality of the learning. The students were found to move away from a perspective focused only on their own needs, toward a multifaceted, system-oriented perspective that treats the ecosystem as a whole.”

The entry is a researcher's interpretation and not an independent measure, written by the person who taught the lessons; the expectancy risk it carries is addressed under Limitations.

Instructional refinement therefore occurred through four immediate adaptations and one prospective modification; the cumulative integration of earlier concepts in the final week belonged to the original design and is reported separately.

### 3.3 Changes in students' expressed sustainability conceptions

Analysis of the post-instruction sustainability conception task showed a clear broadening of students' expressed understandings. All 31 students completed the final task (denominator = 31), and the final dataset yielded 121 coded ideas in total (*M* = 3.90 per student) and the initial dataset 61 (*M* = 1.97); because each dataset was coded in its own frame, the comparable cross-time figures are those reported in Table 5. In contrast to the initial responses, which were largely centered on everyday routines, continuity, and isolated environmental behaviors, the final responses reflected four broader themes, set out with their code frequencies in Table 7: conscious consumption and resource management, nature protection and environmental awareness, social responsibility and ethical values, and future-oriented living.

**Table 7:** Students' final conceptions of sustainability.

Theme	Codes	*f*
Conscious consumption and resource management	Conserving resources such as water, electricity, and paper	16
	Preventing waste of food and materials	11
	Spending according to need	8
Nature protection and environmental awareness	Waste separation and recycling	19
	Protecting natural life and living organisms	13
	Preventing environmental pollution	9
Social responsibility and ethical values	Helping and social solidarity	8
	Awareness of rights, justice, and equality	5
	Being a sensitive and responsible person	10
Future-oriented living	Considering future generations	9
	Sensitivity to global problems	6
	Viewing sustainability as a way of life	7

The most frequently expressed code was waste separation and recycling (*f* = 19), followed by resource conservation (*f* = 16) and protection of natural life and living organisms (*f* = 13). These findings indicate that students increasingly associated sustainability with concrete, action-oriented ideas; such references indicate action-oriented thinking expressed in writing rather than observed behavior or demonstrated action competence. Resource use also became more explicit, with students referring to water, electricity, paper, food, and material consumption.

Social and ethical content also appeared, students referring to helping others, solidarity, justice, equality and being a sensitive person, so that sustainability was no longer expressed primarily as an environmental matter.

Taken together, these group-level patterns indicate a broadening from routine-based conceptions toward a wider range of expressed environmental, social, ethical, action-related, and future-oriented ideas. A larger number of expressed concepts, however, demonstrates broader content rather than, by itself, coherent integration or conceptual reorganization. Evidence bearing on integration is therefore examined through the matched participant-level analysis below, which also tests whether the broadening survives when response length is taken into account, and through the dialogue episodes. The development was not uniform across all students, nor does it imply mastery of sustainability literacy.

### 3.4 Matched participant-level trajectories

Table 5 reports the recoded data. Because the same six-domain codebook was applied at both time points, every row supports a direct cross-time comparison. Three patterns are visible. The everyday-routine domain, expressed by 23 of the 31 students before instruction, appears in none of the final responses. Environmental content rose from 10 students to 30, and social and ethical content from 4 to 23. Resource use and future-oriented outlook, absent from every initial response, appear in 25 and 14 final responses, respectively. The mean number of domains expressed per student rose from 1.58 to 3.19.

The continuity domain behaves differently from the rest, and its pattern bears directly on whether students replaced their earlier understanding or added to it. Twelve students framed sustainability as something being kept going before instruction; seven did so afterwards. That net decline conceals three distinct movements. Three students (S02, S07, S27) retained the continuity frame and filled it with sustainability content: S27 moved from “It means that life and the things we do continue” to “A life in which recycling is done, in which people are not separated into women and men, and in which everyone applies this in daily life.” Four students (S12, S18, S23, S30) expressed continuity only in their final responses, having not used it initially: “Sustainability is not a one-off thing, it should be a way of living” (S23); “Sustainability is continuing to live like this” (S30). Nine students dropped the frame altogether. The idea of continuing is therefore neither simply abandoned nor simply retained; in a minority of cases it is reoccupied, and in four cases it is acquired as a way of describing a practice rather than a routine.

At the level of individual trajectories, no student's set of domains remained unchanged. Twenty-six of the 31 followed a replacement pattern, adding domains while ceasing to express at least one they had used before; five added domains without losing any; none contracted. The dominant movement in this classroom was therefore not accumulation but substitution—the everyday framing giving way to environmental, social, resource-related and future-oriented content. Participant-level detail for all 31 students is given in Supplementary Table S3.

Response length was examined for the same reason. Initial responses contained 251 words in total (*M* = 8.1 per student) and final responses 535 (*M* = 17.3). Because the two inductive codebooks are not comparable across time, density was calculated from the common codebook: the initial responses expressed 49 domains in total and the final responses 99. Words therefore grew by a factor of 2.13 and expressed domains by 2.02, so that domain density was essentially unchanged and slightly lower after instruction (19.5 vs. 18.5 domains per 100 words). Interpreted strictly, the increase in the *number* of expressed domains is proportional to the increase in length and cannot by itself be read as conceptual enrichment. What length does not explain is the change in the *kind* of content: the domain shifts reported above concern which students expressed a given kind of idea, not how much they wrote.

Three matched cases illustrate the range. S05 moved from “*Getting ready in the morning and going to school, eating, and doing homework”* to “*Using only as much water and paper as we need, not wasting food, protecting trees and animals, helping those in difficulty; because people will live in the future too,”* adding four domains and offering a reason. S27, quoted above, kept the continuity frame and replaced its content. By contrast S15 moved from “*Eating, drinking water, and continuing to live by meeting our daily needs”* to “*Not turning the tap on unnecessarily, putting paper and plastics in recycling, protecting trees and animals”*—gaining environmental and resource-related content but nothing social, ethical, future-oriented or relational, with the smallest length increase of the class (10 to 12 words). S19 followed the same narrow pattern. Cases of this kind caution against a uniform narrative of development.

### 3.5 A critical learning episode: from visible cleanliness to biological safety

The third-week activity on SDG 3 and SDG 6 provided the clearest example of conceptual reconsideration in the process. Asked before the filtration activity how they would clean water taken from a puddle, many students associated safety with the removal of visible impurities.

For example, one student stated:

“First, I would remove the sand or leaves from the water, clean it, and drink it.”

Such responses indicated that students generally understood clean water in physical terms and had limited awareness of invisible biological risks. Based on the assessment criteria, 5 responses were classified as qualified, 13 as intermediate, and 9 as limited; four students were absent during this specific activity.

Responses at the other two levels illustrate what those categories contained. A qualified response combined a physical barrier with a biological step: “*I would tear off a huge leaf and put it over the water in the glass and cook it, and the dirty water would come out, and then I would also boil the clean water… and then I would drink it.”* A limited response offered no treatment at all: “*I would tell people to keep the water clean, and I would drink it.”* The response quoted above, which stopped at removing sand and leaves, is typical of the intermediate category.

The second of the three constructs distinguished in the Method can be bounded from these data. Thirteen of the 27 students present explicitly treated the removal of visible material as sufficient for drinking, so at most 14 responses are compatible with recognizing that visual clarity does not by itself imply potability, and of those only 5 named a step addressing what cannot be seen. No written instrument was administered after the activity to re-assess this construct, so evidence that it shifted comes from the classroom dialogue reported below and is weaker than the evidence for the first and third constructs.

The researchers therefore refined the lesson by explicitly distinguishing between visible cleanliness and biological safety. Following the filtration experiment, classroom dialogue revealed a gradual reconsideration of students' initial assumptions:

**Teacher-researcher:** Do you think this water is now safe to drink?

**Student 1:** I think it is, because it looks much cleaner now. […]

**Student 2:** It looks clean, but there may still be invisible things in it. […]

**Student 3:** There may be microbes. They can make us sick. […]

**Student 1:** Now I understand. Looking clean is not enough; the microbes also have to be removed.

This interaction indicated a shift, within this episode, from appearance-based reasoning toward a more scientifically informed understanding of water safety. Students began to recognize that physical filtration may improve visible water quality without necessarily eliminating microorganisms or other biological hazards.

After the activity, students responded to the question:

“Would the result be the same if only one material had been used in the filter? Why?”

The post-activity responses showed stronger reasoning concerning the functional role of filtration materials. Fourteen responses were classified as qualified, eight as intermediate, and five as limited, while four students were absent. One student explained:

“The result would not be the same because each material has a purpose. If there were no charcoal, we could not filter the water properly.”

The two remaining post-activity levels are illustrated by the following responses. An intermediate response predicted the change without justifying it: “*No, it would not.”* A limited response neither predicted the change nor justified it: “*It would be the same because this one is better.”* The latter reproduces, after the activity, the very reasoning the lesson had set out to unsettle.

The two tasks were not identical and are not treated as equivalent pre- and post-tests; they provide complementary evidence within one learning episode, whose significance lies in the visible move from the assumption that clean-looking water is safe toward reasoning about invisible contamination and the functional logic of multi-stage filtration.

This episode also illustrates the central logic of the action research process. Student responses revealed a conceptual limitation; the researchers reflected on this evidence and deepened the instructional process; and subsequent dialogue and written responses indicated conceptual reconsideration. What the episode demonstrates is immediate, scaffolded reconsideration within a supported classroom context; durable conceptual change cannot be established from this episode alone, because no delayed or transfer measures were collected. Thus, the clean-water activity exemplified how classroom evidence, pedagogical reflection, and instructional refinement interacted within a single learning episode.

### 3.6 Classroom dialogue and artifacts as contextual evidence of broadening sustainability conceptions

Classroom dialogue and student artifacts across different weeks provided contextual evidence that students' expressed sustainability-related reasoning extended beyond isolated environmental behaviors toward more interconnected social, ecological, economic, and ethical understandings. The episodes below are drawn from verbatim transcripts of audio-recorded lessons and from student worksheets, and were selected according to the criteria described in the Method (Classroom Observations and Dialogue). Rather than functioning merely as illustrative quotations, these interactions show how students articulated prior ideas, responded to questions, connected sustainability issues, and progressively constructed more complex explanations.

During the first week, which focused on SDG 1 (No Poverty) and SDG 2 (Zero Hunger), students initially examined why people might experience hunger even in places with natural resources. The following exchange illustrates how the discussion developed from a simple question about food scarcity toward relationships among climate, agriculture, food availability, and economic access:

**Teacher-researcher:** There are rivers, fields, and forests in this country. Why, then, might people still be hungry?

**Student 1:** Maybe there is a river, but they cannot use the water.

**Teacher-researcher:** Why might they be unable to use it?

**Student 2:** Maybe because of drought. If there is no water in the river, farmers cannot irrigate. […]

**Student 3:** My grandmother's cucumbers did not grow because there was no rain.

**Student 4:** Our tomatoes also dried in the summer. Then my mother bought them from the market, but they were expensive. […]

**Student 7:** If there is no water, fruit and vegetables do not grow. Then we cannot eat and we become hungry.

This interaction indicated emerging relational reasoning among drought, agricultural production, food supply, prices, and hunger. Students moved from treating hunger as an isolated lack of food toward recognizing the interdependence of environmental and socioeconomic processes.

In the same week, written work from the station-technique task extended this reasoning from food to rights more broadly, one student observing that “*because there is so much hunger we become weak and health problems appear.”* Later in the same discussion students reframed scarcity as a distribution problem—“*some take all of it and there is none left for the others”*—and, after one student offered the analogy of a single shared toy, another concluded that the difficulty lay “*not in the toy… but in not sharing.”*

During the second week, which addressed SDG 13 (Climate Action), SDG 14 (Life Below Water), and SDG 15 (Life on Land), students completed written role-taking tasks in which they wrote from the perspective of a living creature affected by environmental degradation. These artifacts show pollution framed not as an aesthetic problem but as a threat to vital functions, and several contain explicit causal chains: writing as a dolphin, one student stated that “*the reason for dying is that because of the rubbish my breathing decreases and I cannot get oxygen,”* while a student writing as a bird linked discarded batteries to infection and lost nesting sites. Recurring expressions such as “*I would be sad”* and “*I would be cross with people”* indicate that ecological harm was expressed in ethical as well as descriptive terms. Further examples are given in Supplementary Table S1.

A similar expansion was observed in the fourth week during the activity on SDG 17 (Partnerships for the Goals). Students were asked how the international community could assist a fictional country experiencing multiple crises. One student initially proposed that a militarily powerful country should solve the problem alone. Through guided questioning, however, students gradually recognized that ending armed conflict would not automatically resolve hunger, unemployment, infrastructure problems, or social instability:

**Teacher-researcher:** If only a militarily powerful country helps, will all the country's problems be solved? What about hunger, for example?

**Student 3:** No. The war may end, but people may still be hungry.

**Teacher-researcher:** Then what else is needed?

**Student 3:** A country that sends money.

**Teacher-researcher:** Would money alone solve unemployment?

**Student 4:** No. People need schools and factories to work. […]

**Student 6:** All countries should help together.

**Student 7:** One with soldiers, one with money, one with technology.

**Student 8:** It cannot be done alone. Partnership is needed.

The dialogue indicates a shift from a single-actor, single-solution perspective toward recognition of complex problems as requiring coordinated contributions involving security, economic resources, technology, education, employment, and international cooperation.

The fifth-week dialogue on quality education, gender equality, and reduced inequalities provided evidence of developing ethical and justice-oriented reasoning. When students were asked how scientific progress might have differed if women had historically had equal opportunities to become scientists, they responded:

**Student 1:** There would have been more scientists because not only men but also women could have worked.

**Student 2:** More things might have been invented. […]

**Teacher-researcher:** Should people's abilities be judged according to whether they are girls or boys?

**Student 5:** No, because everyone can do something if they put their mind to it.

**Student 6:** Sometimes a thousand people cannot discover something, but one person can. That person could be a woman or a man.

**Student 8:** Nobody chooses whether they are born a woman or a man, so it is not fair.

Here, students moved beyond simple statements supporting equality and began to provide reasons based on fairness, equal opportunity, individual potential, and the arbitrary nature of gender-based restrictions. This suggests that students' expressed conceptions were broadening not only in environmental terms but also through ethical reflection on justice and inclusion.

Asked how they would feel in the position of a classmate whose interest in science went unsupported, students moved from principle to concrete action—introducing that classmate to others, ensuring “*other people also saw her talent,”* or, in one case, “*setting up a laboratory for her.”* These are stated intentions rather than observed actions, but they show equality being reasoned about in terms of what makes participation possible.

Not all evidence pointed in the same direction. Even after the third-week activity, five responses remained limited: they neither predicted that the outcome would change nor justified any prediction, as in “*It would be the same because this one is better,”* which reproduces the pre-activity pattern the lesson had targeted. Eight further responses predicted a different outcome but could not explain why, indicating intuitive rather than functional reasoning. Similar unevenness appeared in the conception tasks: the matched analysis reports one student whose environmental content was present initially and absent finally, and two students (S15 and S19) whose expansion was narrow—gaining resource-use and environmental content but no social, ethical, or future-oriented element (see Matched Participant-Level Trajectories). These cases qualify the overall pattern and informed the interpretation offered in the Discussion.

Taken together, these selected episodes and artifacts indicate that students increasingly connected environmental, social, economic, and ethical dimensions of sustainability in classroom talk and written work, recognizing relationships among environmental change, food systems, economic consequences, international cooperation, and social justice. Because the episodes were purposively selected according to the criteria described in the Method, they provide contextual evidence of the forms of relational reasoning that emerged in this classroom rather than a comprehensive measurement of systems thinking across all students.

## 4 Discussion

### 4.1 From everyday thinking to broader expressed sustainability conceptions

The findings revealed a substantial broadening in students' expressed conceptions of sustainability over the 8-week process. Beforehand, students interpreted sustainability through familiar routines, the literal meaning of continuity and isolated environmental behaviors—conceptions consistent with previous research showing that young learners hold fragmented, context-dependent understandings (Walshe, 2008; Petrou and Korfiatis, 2022; Toma et al., 2024). Afterwards their responses referred to resource conservation, responsible consumption, biodiversity protection, solidarity, justice, equality, global challenges and future generations—changes that the recoded matched analysis registers as cross-time movement and not merely as features of the final responses (Berglund and Gericke, 2016). This is the kind of movement that contemporary accounts of sustainability literacy describe, and GreenComp offers a useful interpretive frame for it (Bianchi et al., 2022; Vesterinen and Ratinen, 2024; Toma et al., 2024), though the responses show early developmental tendencies and not acquired competencies.

Future-oriented ideas were among the clearest additions: none of the initial responses expressed them, whereas 14 of the final responses did (Bianchi et al., 2022; Vesterinen and Ratinen, 2024). Whether these changes reflect genuine conceptual reorganization or primarily the addition of recently taught concepts is a central interpretive question. The matched analysis allows this question to be addressed more precisely than group frequencies alone. Two findings pull in different directions. On the one hand, density did not increase: final responses contained roughly twice as many words and roughly twice as many expressed domains, so the growth is proportional to length and cannot be read as enrichment. On the other hand, the composition of what students expressed changed in ways length cannot explain—environmental content rose from 10 to 30 students and social/ethical content from 4 to 23—and several individual trajectories look reorganizational rather than merely additive. The clearest instances are students whose wording retains the Turkish word's literal sense of “continuing” while filling it with new content, as when a student who had defined sustainability as “life and the things we do continue” finally described “a life in which recycling is done, in which people are not separated into women and men, and in which everyone applies this in daily life.” Read as text, movement of this kind looks reinterpretative rather than additive, although the coding frame registers only the loss of one code and the addition of others. Even so, in the absence of delayed and transfer tasks, and given that one student's environmental content disappeared rather than accumulated, the overall pattern is most accurately described as a broadening of expressed conceptions with emerging, uneven integration rather than as demonstrated conceptual reorganization.

### 4.2 Relational reasoning in selected dialogue episodes

Three features of the episodes deserve comment, and each should be read as a property of particular exchanges rather than of the class. First, students constructed multi-step causal chains: drought to irrigation to production to prices to hunger in the first week, and waste to reduced oxygen to the death of a marine animal in the second. Second, they came to attribute complementary roles to different actors, moving from the proposal that one powerful country should resolve a crisis to the recognition that security, money and technology are separate contributions. Third, in the final design task they brought previously separate topics together in a single product. The study does not claim that fourth-grade students acquired mature systems-thinking competence, nor was systems thinking measured as a general competence; these are the specific relational features the data contain (Assaraf and Orion, 2010; Mambrey et al., 2022).

### 4.3 The role of classroom dialogue and iterative instructional refinement

Ethical and social reasoning developed in the same episodic way. In the gender-equality discussion students moved from expressing support for equality to justifying it through fairness, equal opportunity and the arbitrariness of the circumstances of birth, and then to proposing concrete acts of support; in the partnership scenario they moved from a single-actor solution to shared responsibility. What these exchanges have in common is that the reasoning was elicited by questions that asked for justification rather than assent, which is also what the cycle log records as the fifth-week adaptation. The broadening documented in the conception tasks therefore appears alongside, and is contextualized by, a particular kind of classroom talk—one in which students' initial formulations were made explicit and then queried (Rudsberg and Öhman, 2010; Rodnes and Dolonen, 2023; Reffhaug et al., 2024; Haggström, 2022).

The findings are therefore consistent with the view that sustainability education involves not only what is taught but also how systematically teachers attend to students' reasoning and use classroom evidence to guide subsequent instruction, although a single classroom cannot establish this. Classroom action research offers a workable framework for organizing that attention (Haggström, 2022).

### 4.4 Interpreting the findings through greencomp and education for sustainable development

Education for Sustainable Development provides the pedagogical orientation for this interpretation; GreenComp, used here solely as an interpretive lens and not as an assessment framework, describes how learners develop sustainability-related values, systems thinking, future orientation, and responsible action (UNESCO, 2020; Bianchi et al., 2022; Vesterinen and Ratinen, 2024).

Viewed selectively through GreenComp's four competence areas (Bianchi et al., 2022), the evidence is uneven, and it is important to distinguish what the data can and cannot support. Expressions related to embodying sustainability values—fairness, equality, responsibility, respect for nature—and to embracing complexity—linking environmental, social, and economic issues—were relatively well represented, the latter in selected dialogue episodes and artifacts. Envisioning sustainable futures appeared in a weaker, emergent form, mainly as references to future generations and long-term consequences. By contrast, acting for sustainability cannot be established from classroom statements alone: references to recycling, conservation, or helping indicate action-oriented ideas rather than observed behavior, and no behavioral measures were collected (Sass et al., 2020).

These findings should be interpreted developmentally rather than as evidence that students had fully acquired GreenComp competencies. What the data show is narrower: within one classroom, students' written and spoken accounts of sustainability came to include a wider range of environmental, social, and ethical content. Whether broadening of this kind later consolidates into the values, systems thinking, future orientation, and responsible action these frameworks describe is a question for longitudinal research (Bianchi et al., 2022; Vesterinen and Ratinen, 2024).

### 4.5 Contribution to the literature and educational implications

The study contributes to sustainability education research by documenting how students' expressed sustainability conceptions broadened during an iterative, evidence-informed process, rather than by asking only whether an intervention was effective (Haggström, 2022; Petrou and Korfiatis, 2022; Reffhaug et al., 2024). It also shows what a matched, common-codebook analysis can reveal that aggregate frequencies conceal—here, that what students wrote changed by substitution rather than accumulation.

The elements of this account—ESD as orientation, the SDGs as interdisciplinary contexts, GreenComp as a secondary lens, and classroom action research as the method of refinement—are offered as an illustrative framework for further testing rather than as a validated one (UNESCO, 2020; Kioupi and Voulvoulis, 2019; Bianchi et al., 2022; Haggström, 2022).

### 4.6 From expressed conceptions to long-term environmental commitment and nature connectedness

This study examined expressed conceptions; it did not measure nature connectedness, environmental values, behavior, or commitment, and no empirical claim is made about such outcomes. Nevertheless, the findings can be positioned in relation to the literature on how durable environmental engagement develops. Research on the childhood origins of environmental engagement indicates that experiences and meanings formed during the primary years matter for later environmental attitudes, connectedness, and behavior (Wells and Lekies, 2006; Evans et al., 2018; Rosa et al., 2018). Connectedness to nature, together with environmental knowledge, has been identified as a central correlate of ecological behavior in children (Otto and Pensini, 2017), and educational interventions can strengthen nature connection in childhood, with effects that appear more durable before approximately age 11 (Liefländer et al., 2013; Barrable and Booth, 2020)—precisely the age band of the present participants.

One feature of the present data is worth noting in this light, without overstating it: the second-week role-taking artifacts show students writing about environmental harm in affective as well as factual terms—difficulty breathing, losing a nest, indignation at pollution—and affective engagement is the register in which nature connection is ordinarily described. This is an expressed meaning, not a measured disposition. The instructional process documented here was classroom-based and included no direct contact with nature, so the relation between such expression and any durable bond with the natural world is precisely what remains untested. Combining SDG-based conceptual work with direct nature experience, and following connectedness and commitment longitudinally, is accordingly identified as a priority for subsequent research rather than as an achievement of this study.

### 4.7 Alternative explanations and language considerations

The observed changes admit several credible alternative explanations that limit causal attribution. First, students were directly exposed to the taught content for 8 weeks, so post-instruction responses may partly echo recently taught vocabulary rather than reorganized understanding. Second, the same conception prompt was repeated, and familiarity with the task may have facilitated fuller answers. Third, final responses were more than twice as long (*M* = 8.1 to 17.3 words), and the number of domains expressed grew by almost exactly the same factor, leaving domain density essentially unchanged and in fact marginally lower (19.5 to 18.5 domains per 100 words); this is the alternative explanation the present data support most directly, and it is the reason the argument rests on which kinds of content students expressed rather than on how many ideas they produced. Fourth, social-desirability pressures and the visible investment of the researcher-teacher in sustainability may have encouraged answers students perceived as valued—a risk that is particularly relevant because the person who taught the lessons also collected and interpreted much of the evidence. Fifth, ordinary maturation and concurrent curricular learning over the 2-month period cannot be ruled out. Finally, the purposive selection of dialogue episodes risks over representing successful interactions, which is why counter-examples and a limited-change case are reported. These alternatives do not invalidate the observed broadening of expressed conceptions, but they do mean that the SDG-based process should be understood as a plausible pedagogical accompaniment of these changes, not as a demonstrated cause.

A related interpretive consideration concerns language. The study was conducted in Turkish, and the Turkish term for sustainability, *sürdürülebilirlik*, is morphologically transparent: it derives from *sürdürmek* (“to continue, to maintain”). Fourth-grade students encountering the term may reasonably parse it as “the ability to keep something going,” which can account for the prominence of literal continuity answers in the initial task. Such answers may therefore reflect lexical unfamiliarity with the specialized concept and not conceptual limitation, and the initial coding categories should be read with this possibility in mind. Conversely, the broader content of the final responses incorporated vocabulary introduced during instruction, which is expected in any teaching study but complicates the distinction between lexical and conceptual growth. Cross-linguistic replication would help disentangle these possibilities.

### 4.8 Limitations

This study has important limitations. It was conducted in a single intact class in one school, and the findings are context-bound rather than generalizable. The researchers designed, taught, and analyzed the intervention; despite structured observation by the observing researcher, audio recording, reflective journaling, and consensus procedures, expectancy effects cannot be excluded, and students' awareness of the researcher-teacher's interest in sustainability makes social-desirability effects plausible. Although dialogue was audio-recorded and transcribed verbatim, episodes were purposively selected, and a risk of emphasizing especially positive episodes remains; counter-examples were therefore actively sought and reported. Translation of excerpts from Turkish into English, the morphological transparency of *sürdürülebilirlik*, and the repetition of the same conception prompt complicate interpretation, as do differences in response length between the two tasks. The water-activity tasks were non-equivalent and are not a pre/post measure. No delayed, transfer, or behavioral measures were collected, so durability, generalization to new contexts, and action cannot be established. The matched analysis carries its own limitations. The common six-domain codebook was constructed after the inductive coding rather than specified in advance, and although it was applied identically to both sets of scripts by two coders working independently, a framework fixed before data collection would be preferable. The analysis also rests on brief written responses from young children-−251 words in total before instruction and 535 after—which constrains the inferences any single source can support, and the substitution pattern it reveals concerns what students chose to write on one occasion rather than the whole of what they understood. The practical implications that survive these limitations are correspondingly modest and process-oriented: eliciting students' initial meanings, using prompts about causes, consequences, actors, time horizons, and trade-offs, revisiting misconceptions explicitly, and maintaining a structured reflection log of pedagogical decisions.

## 5 Conclusions

This study traced primary school students' expressed sustainability conceptions through an eight-week interdisciplinary SDG-based classroom action research process. Within this classroom, post-instruction responses displayed a broader and more multidimensional range of expressed sustainability conceptions than initial responses: whereas initial responses centered on everyday routines, literal continuity, and isolated environmental behaviors, final responses referred to resource conservation, responsible consumption, biodiversity, social justice, equality, collective responsibility, and future generations. In the matched recoded comparison, environmental content rose from 10 to 30 of the 31 students and social and ethical content from 4 to 23, everyday-routine framing disappeared, and domain density did not rise—so the claim the data support concerns the kind of content students expressed, not its quantity.

These conclusions concern students' expressed conceptual understandings—what students wrote and said—rather than stable values, competences, behavior, agency, or civic engagement, none of which were measured. The pattern is most accurately described as conceptual development: a documented broadening of expressed conceptions with emerging signs of integration, rather than a completed conceptual transformation.

The study also highlights the importance of responsive pedagogy. Classroom dialogue, students' emerging ideas, misconceptions, and learning products informed the instructional decisions recorded for each cycle. This illustrates how classroom action research can organize responsive sustainability teaching in primary education.

The SDGs functioned as interdisciplinary learning contexts that connected environmental, social, ethical, and economic dimensions of sustainability. The final week's design task, in which groups brought earlier topics together in a single plan, carried the same integrative intent.

The limitations set out in the Discussion bound these conclusions directly: the single-class design, the dual researcher-teacher role, expectancy and social-desirability effects, language and prompt-repetition issues, and the absence of delayed, transfer, or behavioral measures. Accordingly, the study presents an illustrative instructional and analytical approach for further testing, not a validated model.

Future research should follow matched qualitative trajectories over longer periods; include delayed and transfer tasks to examine durability and generalization; conduct comparative action research across classrooms, age groups, and cultural-linguistic settings; employ independent observation and coding; and combine conception measures with instruments that distinguish conceptual understanding from nature connectedness, environmental commitment, values, and behavior. Studies that pair SDG-based conceptual work with direct nature experiences would be especially informative about whether broadened conceptions help lay the foundation for long-term environmental commitment.

Overall, within this classroom, an interdisciplinary, dialogic and iteratively refined SDG-based process was accompanied by a documented broadening of students' expressed sustainability conceptions. If such foundations are consolidated through continued nature contact and value-supportive experiences, they may contribute, over time, to the long-term environmental commitment that sustainability ultimately requires—a proposition this study motivates but does not test.

## Data Availability

The raw data supporting the conclusions of this article will be made available by the authors, without undue reservation.
